# A Digital Twin Framework for Visual Perception in Electrical Substations Under Dynamic Environmental Conditions

**DOI:** 10.3390/s25185689

**Published:** 2025-09-12

**Authors:** Tiago Trindade Ribeiro, Andre Gustavo Scolari Conceição, Leonardo de Mello Honório, Iago Zanuti Biundini, Celso Moreira Lima

**Affiliations:** 1 Department of Electrical and Computer Engineering, Federal University of Bahia, R. Prof. Aristídes Novis 2, Federação, Salvador 40210-630, BA, Brazil; andre.gustavo@ufba.br; 2 INESC P&D Brasil, R. José Caballero 15, Gonzaga, Santos 11055-300, SP, Brazil; leonardo.honorio@ufjf.br (L.d.M.H.); iago.biundini@gmail.com (I.Z.B.); 3 Department of Electrical Engineering, Federal University of Juiz de Fora, R. José Lourenço Kelmer, São Pedro, Juiz de Fora 36036-900, MG, Brazil; 4 Argo Energia, R. Tabapuã, 841-51-Itaim Bibi, São Paulo 04538-132, SP, Brazil; celso.lima@argoenergia.com.br

**Keywords:** digital twin, visual perception, sensor simulation, gazebo, electrical substations, Render-In-The-Loop, computer vision, robotics

## Abstract

Electrical power substations are visually complex and safety-critical environments with restricted access and highly variable lighting; a digital twin (DT) framework provides a controlled and repeatable context for developing and validating vision-based inspections. This paper presents a novel sensor-centric DT framework that combines accurate 3D substation geometry with physically based lighting dynamics (realistic diurnal variation, interactive sun-pose control) and representative optical imperfections. A Render-In-The-Loop (RITL) pipeline generates synthetic datasets with configurable sensor models, variable lighting, and time-dependent material responses, including dynamic object properties. A representative case study evaluates how well the framework reproduces the typical perceptual challenges of substation inspection, and the results indicate strong potential to support the development, testing, and benchmarking of robotic perception algorithms in large-scale, complex environments. This research is useful to utility operators and asset management teams, robotics/computer vision researchers, and inspection and sensor platform vendors by enabling the generation of reproducible datasets, benchmarking, and pre-deployment testing.

## 1. Introduction

Autonomous inspection of electrical substations is an emerging field with significant potential to improve maintenance efficiency, operational safety, and inspection frequency of critical infrastructures [1,2]. Power transmission substations represent a particularly challenging environment for robotic perception due to their complex and large-scale structural geometries, consisting of metallic structures, cables, insulators, and various high-voltage equipment [3].

**Figure 1:**
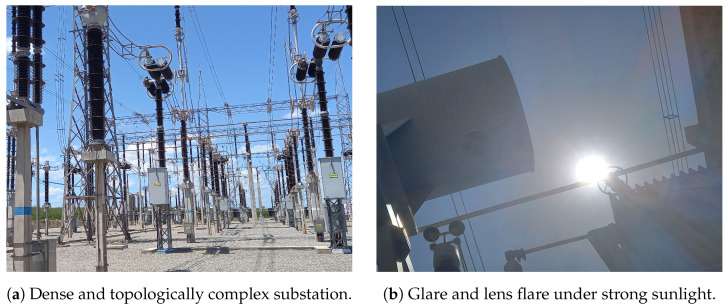
Environmental complexity in outdoor substation and visual phenomena scenarios.

These environments often encompass large outdoor areas with non-planar navigation surfaces, where the combination of high-dimensional variability and complex, densely populated 3D layouts imposes stringent demands on the accuracy and robustness of visual perception systems (Figure 1a). Furthermore, the presence of electromagnetic interference (EMI) zones near high-voltage conductors, transformers, and switching devices introduces additional complexity by limiting the applicability of some inspection solutions [4,5,6,7].

Sensors may exhibit degraded performance or increased noise levels in these regions, which can additionally be hazardous to humans, highlighting the importance of realistic simulation environments for a priori testing and validation [8,9]. Lighting conditions further aggravate perceptual challenges since substations are typically inspected under variable natural lighting, causing effects such as glare, saturation, and reflections (Figure 1b) [10,11]. These conditions pose challenges for reliable RGB-based perception and motivate the use of realistic simulation.

Realistic simulation frameworks are essential for accelerating the development and evaluation of perception and navigation algorithms, especially in applying deep reinforcement learning (DRL) strategies [12,13,14], where robust policy learning relies on extensive interaction across varied scenarios.

The ability to systematically control environmental parameters such as lighting, sensor characteristics, and object dynamics is essential not only for generating consistent data but, above all, for evaluating and improving perception systems designed to operate in adverse, complex, and potentially harmful conditions for human presence. Realistic simulation environments allow testing inspection algorithms in a safe and controlled manner, anticipating challenges that would be difficult to replicate in the field.

To meet these needs, based on the proposal of Ribeiro et al. [15], this article proposes a realistic simulation framework for visual perception in electrical power transmission substations, combining accurate 3D modeling of the substation geometry with physics-based lighting dynamics, capable of modifying the sun-pose at runtime and of including imperfections typical of visual systems, such as obstruction, glare, reflections, and image overflow.

A Render-In-The-Loop (RITL) pipeline [16] allows the generation of synthetic datasets with configurable sensor models and dynamic environmental properties. In the context of autonomous inspections in power transmission substations, this approach is especially advantageous, as it allows the realistic simulation of natural lighting variations, reflections on metal components, interference from structural shadows, and partial occlusions caused by buildings or equipment.

By enabling the testing of various onboard sensor configurations under extreme conditions, RITL helps anticipate the perceptual challenges inspection robots may face in real environments. These features are particularly beneficial for the robust training of DRL algorithms and the reliable validation of navigation and anomaly detection strategies.

Recent work has advanced robotics and vision simulation along several fronts. Hoskere et al. [17] introduced a 3D synthetic environment that couples physics-based graphics with finite-element modeling to benchmark autonomous inspection. Schraml et al. [18] showed that camera pose, lighting, and noise parameters in synthetic renderings notably affect AI performance for defect detection. A related contribution presents a photorealistic simulation framework that generates datasets across varied times of day and weather conditions, enabling more rigorous testing under dynamic environments [19].

Complementary to these advances, our contribution is substation-specific and sensor-centric, providing a digital-twin, RITL-based framework with a graph formalism that ties physical subsystem states to rendering attributes, supports runtime sun-pose control consistent with site geometry, and targets perception challenges unique to high-voltage substations (e.g., glare, specular metals, complex layouts). Prior works do not offer this combination of substation focus, graph-based DT/RITL coupling, and quantitative perceptual characterization.

We validate the proposed framework through a case study focused on visually monitoring line shunt reactors, essential equipment for reactive power control and voltage stability in transmission systems. The experiment simulates the inspection of three oil level indicators using a photometric model based on the Samsung Galaxy S23 camera (Samsung Electronics Co., Ltd., Suwon, Republic of Korea). Through an RITL pipeline, the framework reproduces variable natural lighting effects and reflections on metal surfaces. It includes the dynamic update, at runtime, of the visual indicators.

By integrating RITL with mathematical modeling, we create a powerful digital-twin environment that not only reproduces realistic visual and physical behaviors of the inspected equipment but also maintains a synchronized virtual replica throughout its operational lifecycle, enabling real-world case studies and optimizations across the entire power system [6,20].

This paper demonstrates the potential of the RITL framework to support the development, testing, and benchmarking of perception algorithms aimed at robotic inspection of power substations. By offering a controlled and reproducible environment, supporting dynamic variation of environmental conditions, and providing real-time visual updates, the framework allows us to accurately evaluate the performance of learning models in challenging scenarios, such as abrupt changes in lighting, specular reflections, and changes in equipment states.

The simulation framework enables isolated validation of sensors and algorithms and safe testing of visual perception–based navigation and decision-making strategies in critical environments within the power sector.

The central research questions answered in this proposal are summarized below:

RQ1: Fidelity: To what extent can a sensor-centric digital twin with physically based lighting (diurnal variation, controllable sun-pose/irradiance) and representative optical effects reproduce the perceptual challenges typical of substation inspection?
RQ2: Dataset generation: Can an RITL pipeline produce controllable and repeatable synthetic datasets with configurable sensor models, variable lighting, and time-dependent material response suitable for perception research?
RQ3: Quantitative characterization: Do objective image-level indicators under changing illumination and scene dynamics capture the expected radiometric/optical behaviors needed to assess visual degradation?
RQ4: Practical utility: Is the resulting environment fit for purpose to support the development, testing, and benchmarking of robotic perception algorithms in large-scale, complex substation settings?

The remainder of this paper is structured as follows: Section 2 reviews relevant literature on simulation and perception in robotic inspection. Section 3 details the architecture of the proposed simulation framework. Section 4 reports the experimental findings based on the case study. Lastly, Section 5 summarizes the main contributions and suggests directions for future research.

## 2. Related Work

Using simulated environments has become essential for developing robotic systems, especially for applications involving operations in complex, large, and potentially harmful environments. Simulation enables the safe, repeatable, and cost-effective evaluation of perception and control algorithms in various scenarios that would otherwise be difficult or dangerous to reproduce in the real world. In recent years, simulation has proven particularly effective in supporting data-driven methods such as deep learning and reinforcement learning, which rely on large amounts of interaction data for training and validation.

Despite significant progress in general-purpose robotic simulation platforms, most available environments focus on domains such as autonomous driving, aerial robotics, or structured indoor environments. In contrast, realistic modeling of outdoor industrial environments such as high-voltage electrical substations remains relatively unexplored. These environments present unique challenges for perception, including complex 3D structures, dynamic daylighting, and electromagnetic interference zones. Furthermore, generating realistic synthetic data under these conditions is critical for developing robust perception pipelines that can operate reliably in the field. The following subsections review related work across three main dimensions: simulation frameworks for robotic perception, RITL techniques used, and the current scarcity of realistic simulated environments for electrical inspection.

### 2.1. Simulated Environments for Robotic Perception

Robot perception research has benefited significantly from advances in simulation platforms. Generic tools such as Gazebo [21], AirSim [22], and CARLA [23]. NVIDIA Isaac Sim [24] offers high-fidelity environments with realistic physics, configurable sensor models, and support for varied environmental conditions, making them fundamental for developing and evaluating robotic applications in navigation, manipulation, and unmanned vehicles.

Recent review studies highlight that simulators such as Gazebo are widely used for their integration with ROS and low computational cost, while CARLA, AirSim, and Isaac Sim stand out for their visual quality and support for urban and outdoor environments with a high degree of realism [25,26]. These capabilities are particularly relevant for applications based on deep reinforcement learning and simulation-to-reality transfer.

However, these tools are general-purpose and require extensive customization in specific scenarios. Currently, no consolidated initiatives or standardized architectures address the realistic simulation of complex environments such as electrical substations. Researchers and developers must address key factors such as complex geometry, dynamic lighting, and the real-time behavior of embedded sensors and actuators in substation equipment when building such simulation environments.

Some recent initiatives have sought to explore more realistic simulations for robotic scenarios, but they still have limitations regarding the generalizability and computational requirements involved. For example, LIMoSim [27] proposes a lightweight and extensible framework for applications in intelligent transportation systems, but it is not directly applicable to complex industrial scenarios such as substations.

Similarly, MORSE [28] offers a modular simulation engine aimed at academic use, but it lacks support for realistic modeling of industrial environments with dynamic lighting and intricate geometry. Although efficient for aerial vehicle applications, the gym-by-bullet-drones simulator [29] remains restricted to a specific niche and does not address robotic perception challenges in outdoor industrial environments.

This gap is also observed in other industrial domains, such as hydroelectric plants, where researchers propose an integrated architecture for X-in-the-Loop simulation [15], focusing on realism. Building on this approach, the present work adopts its core principles as a foundation for modeling and structuring the simulation of electrical substations. The original architecture was adapted to the specific requirements of this new domain in order to create more realistic scenarios aligned with the demands of robotic inspection.

### 2.2. RITL for Perception

RITL methods have emerged as an effective means to generate high-quality, physically realistic synthetic data for perception, control, and other systems. By integrating physically-based rendering with sensor modeling and dynamic scene parameters, RITL enables fine-grained control over the generation of visual information. This approach has roots in previous work that explored synthetic rendering coupled with the training of perception networks [30,31,32].

RITL is well-suited to domains like autonomous driving, aerial robotics, and industrial inspection, where dynamic environments, realistic lighting, and sensor artifacts strongly affect perception. By systematically varying lighting, materials, sensor noise, and equipment states, it enables rigorous training and testing under challenging conditions. This approach also aligns with domain randomization strategies [33], which improve generalization by exposing models to broad variability during training.

Despite the broad applicability and current computational support, the use of RITL, especially for large-scale outdoor industrial inspections such as electrical power substations, remains underexplored. The present work aims to fill this gap by applying RITL to generate realistic perception scenarios for the robotic inspection of substations.

### 2.3. Challenges in Applying RITL to Electrical Substation Inspection

Over the years, robotic inspection of electrical substations has attracted increasing attention [3,34,35]. Several works propose robotic platforms with visual sensors, including RGB-D (Red, Green, and Blue and Depth) and thermal cameras, to perform equipment monitoring, anomaly detection, and visual inspection. However, most of these systems are validated predominantly through field experiments, with limited use of simulation for systematic development and testing.

Existing robotic simulation environments fall short in capturing the unique characteristics of electrical substations. Key challenges, such as dynamic natural lighting (e.g., glare and saturation), highly reflective metallic surfaces, real-time behavior of embedded equipment sensors and actuators, and dense geometric layouts, are often oversimplified or entirely omitted. This lack of realism hinders the development and evaluation of robust perception pipelines suitable for field deployment.

To date, no substation-specific simulation framework reproduces the sensing fidelity required for robotic inspection. Most available simulators target generic infrastructure and do not capture the visual and structural particularities of substations; as a result, perception strategies validated in such simplified settings often fail to transfer to real deployments.

Scientifically, this work addresses four gaps: (G1) lack of substation-specific fidelity, (G2) insufficient modeling of critical optical effects, (G3) limited support for temporal variability (diurnal/operational changes), and (G4) scarcity of controlled, reproducible settings for evaluation and data generation. We tackle these gaps through the following:

- Realistic modeling of the substation environment (addresses G1), with detailed geometry, photometrically plausible materials, and equipment layouts typical of real installations;
- Simulation of critical visual effects (addresses G2), including specular brightness, camera saturation, moving shadows, and dynamic reflections;
- Representation of time-varying operating conditions (addresses G3), encompassing shift changes, day/night transitions, and varying solar incidence;
- Automated generation of synthetic data (addresses G4), with variability control and accurate labeling suitable for supervised and reinforcement training;
- Creation of representative scenarios for evaluating established monitoring techniques (supports G4), facilitating simulation-to-reality transition;
- Support for training technical teams, enabling familiarization with adverse and operational conditions in a safe and reproducible environment.

The proposed simulation framework fills these gaps by providing a digital twin, RITL-based architecture tailored to robotic inspection of electrical substations. It models environmental dynamics and sensor behavior realistically, yielding representative scenarios for evaluating established monitoring techniques. The framework also generates synthetic data with precise control of key variables, supporting training of perception algorithms, including deep reinforcement learning (DRL), and enabling critical, repeatable scenarios for safe operator training.

## 3. Materials and Methods

This work proposes a modular framework for integrated simulation and real-time rendering of visually complex environments. The proposal combines realistic three-dimensional modeling with a formalized graph-based computational architecture, allowing physical elements to adjust their visual representations dynamically during simulation. Although the initial focus is on power transmission substations, the framework is extensible to other domains, such as indoor industrial, urban, or laboratory environments, while maintaining coherence between the simulated physical behavior and its visual representation. The following subsections detail the modeling of the substation elements and the architecture of the proposed real-time rendering pipeline.

### 3.1. Substation Modeling

**Figure 2:**
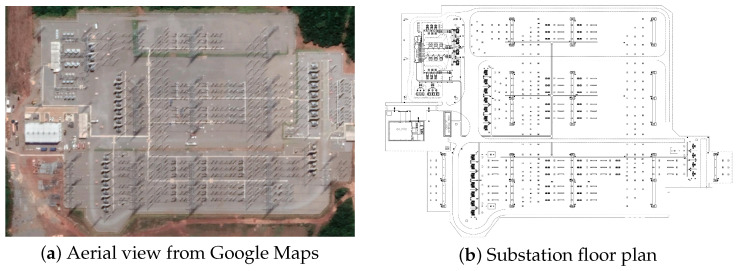
Visual comparison between the aerial view and the floor plan used for 3D reconstruction.

We conducted the three-dimensional modeling of the substation to provide a structured and extensible representation of the main physical components of an electrical power transmission substation. Figure 2 presents the aerial view from Google Maps and the floor plan used as a geometric reference for reconstructing the virtual scenario.

**Figure 3:**
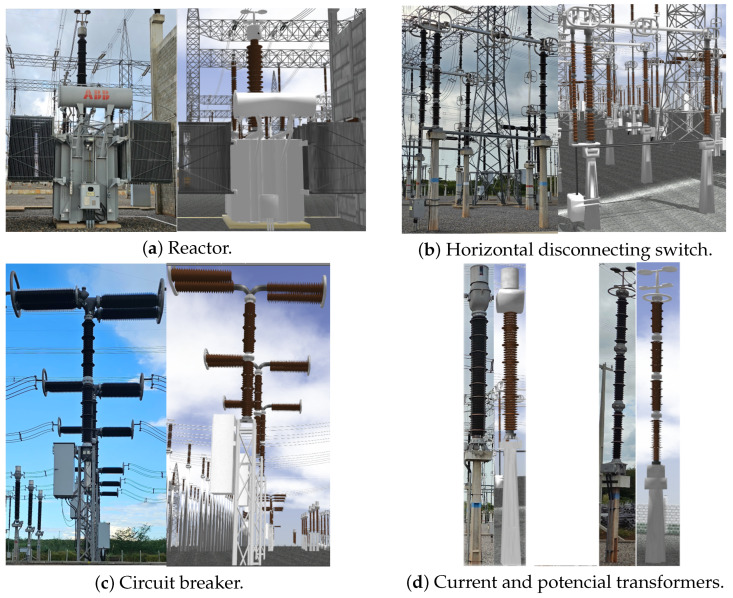
Real equipment (**left**) and corresponding simulated 3D models (**right**).

Using this floor plan, CAD (Computer Aided Design) models, photos, and videos obtained from technical sources, we individually modeled several critical elements for the substation operation in Blender software, including shunt reactors, disconnecting switches, current transformers, and other standard components found in electrical transmission substations. Figure 3 illustrates some of these components.

**Figure 4:**
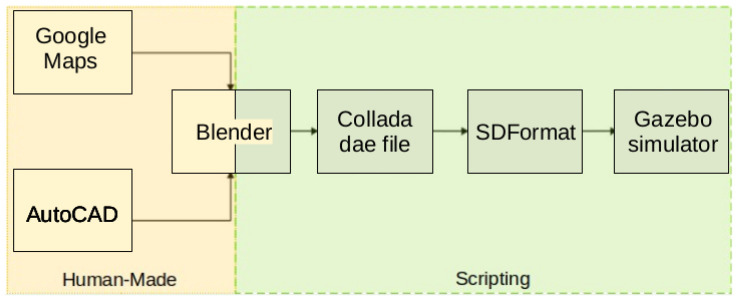
Pipeline for converting and integrating the models into the simulation environment. Adapted from Ribeiro et al. [15].

Integration into Gazebo followed the pipeline in Figure 4, adapted from Ribeiro et al. [15]. Unlike prior uses with few large assets, our scenario has high spatial/structural granularity (hundreds of components), making manual export impractical. We therefore developed custom scripts with the Blender API to automate export to COLLADA (.dae), compatible with SDF (Simulation Description Format), preserving object hierarchies and geometry and ensuring interoperability between modeling and simulation.

The scene was reconstructed in Blender by assembling individual models with their georeferenced coordinates while preserving equipment relative positioning, installation topography, and technical implementation criteria. Because of the complexity of the environment, with about 800 models across three elevation levels, building the simulator scene from a single DAE (Digital Asset Exchange) file was impractical: a monolithic asset increases load time and memory footprint, reduces culling granularity to the whole model, prevents instancing of repeated parts, and precludes asset-level LOD (Level of Detail), per-object physics/collisions, and sensor attachments.

Instead, we integrated the components directly at the SDF file level, referencing each model modularly. Splitting the scene into individual models enables selective loading and updates, clearer organization, and real-time performance suitable for large substation layouts.

**Figure 5:**
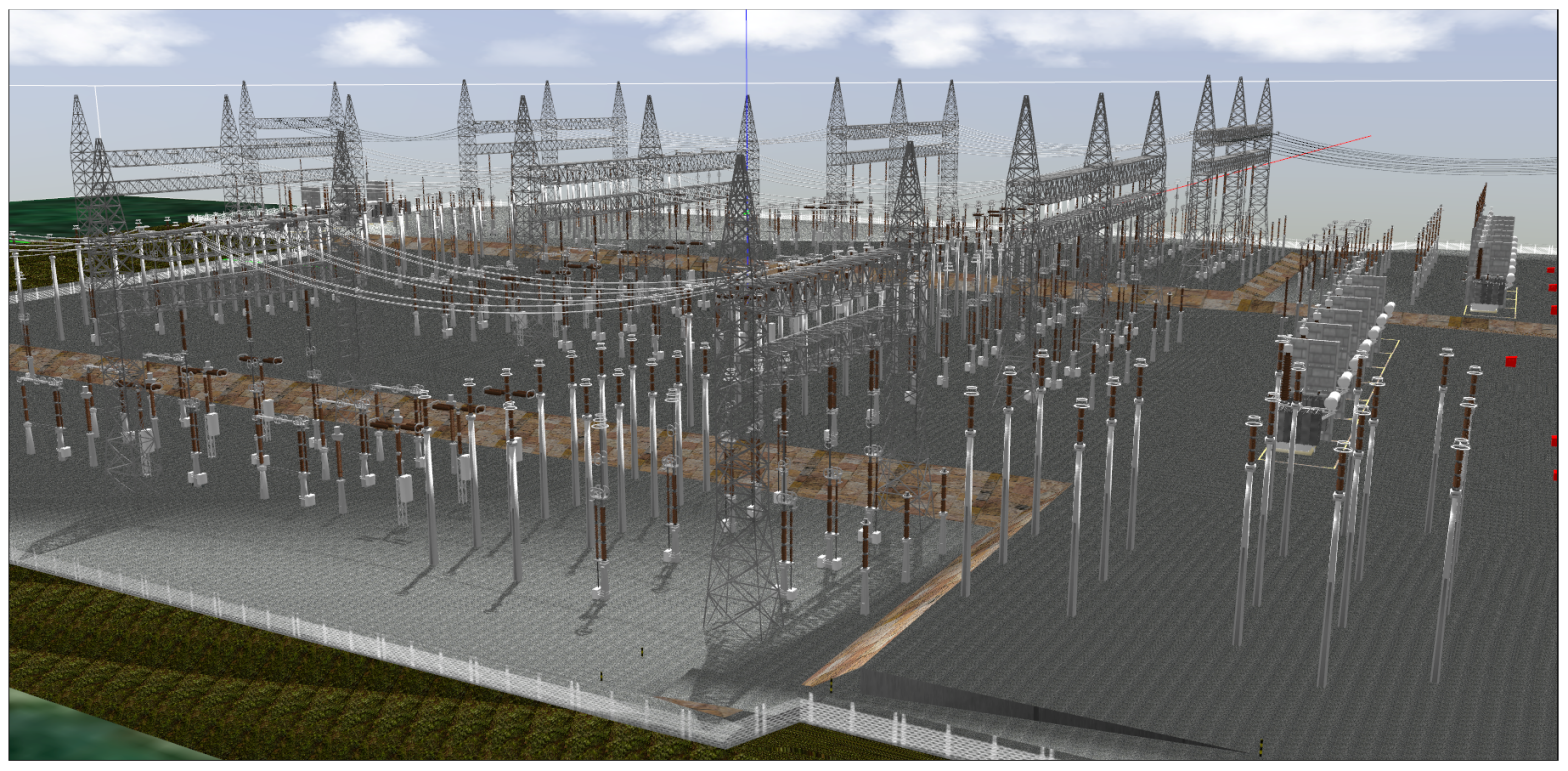
Complete environment modeled and integrated with the Gazebo simulator.

This modular approach promotes model reuse, facilitates specific adjustments, and ensures scalability in the simulation. Figure 5 shows the resulting environment integrated into Gazebo, with all elements positioned to match the actual geometry of the substation, including safety zones and terrain slopes; a video walkthrough is available [36].

Custom scripts were developed with the Blender API to export the models, automating the generation of model directories, the creation of meshes/materials/model files, and the application of vertical offsets reflecting topography levels. These scripts also preserve functional hierarchies, articulation axes, and logical groupings, ensuring compatibility between the visual and physical aspects of the environment. The high dimensionality and structural dynamism of this scenario highlight the need for a formal architecture to organize and manipulate the elements of the system in a scalable, coherent manner, motivating our graph-based approach, as detailed in the next section.

### 3.2. RITL Pipeline

**Figure 6:**
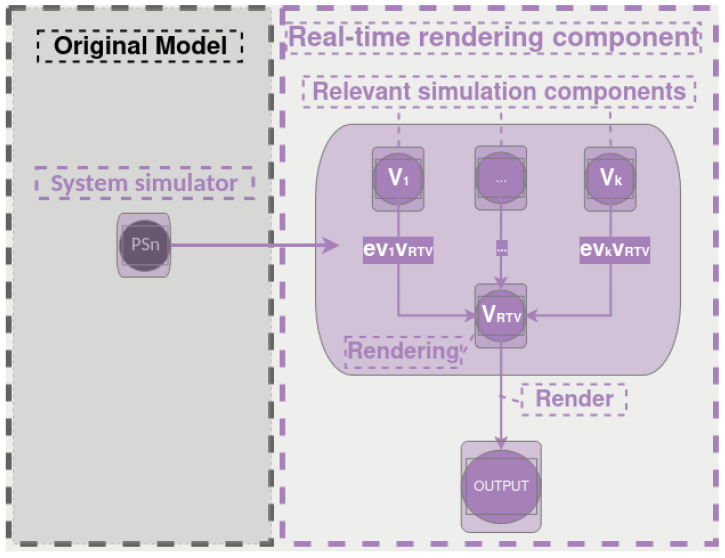
Extension of the model proposed by Ribeiro et al. [15] to support real-time rendering.

This work builds upon the integrated architecture introduced by Ribeiro et al. [15], originally applied to X-in-the-Loop simulations for ASV (Autonomous Surface Vehicles) navigation systems. The overall structure and coupling principles between physical simulation and control frameworks are preserved, with a focused extension on the $PS_{1}\ldots PS_{n}$ modules. A real-time rendering component is introduced to generate visual representations that reflect the internal states of the physical subsystems. This architectural basis is illustrated in Figure 6.

Structurally, the original model is formalized as an undirected and unlabeled graph G=(N,E,W), where N is the set of nodes (including functional components and supernodes), E⊆N×N the edges, and *W* a vector of vertex weights.

We propose a localized extension to the internal structure of the $PS_{1}\ldots PS_{n}$ subgraph, reformulated as a directed and labeled graph G=(V,E,L), defined by the following:

- *V*: functional components of the physical subsystems, including the rendering module;
- E⊆V×V: directed edges representing communication between components;
- L:E→Σ: labeling function encoding semantics such as data types, update rates, or communication protocols.

The rendering module is introduced as a node $v_{\mathrm{RTV}}\in V$, connected to a subset 𝒮⊆V, composed of components whose outputs are relevant for visualization. For each edge $(v_{k},v_{\mathrm{RTV}})\in E$, the labeling function encodes:

(1) $$L\left(v_{k},v_{\mathrm{RTV}}\right)=e_{v_{k}v_{\mathrm{RTV}}}.$$

Each node $v_{i}\in V$ is modeled as a deterministic function:

(2) $$v_{i}:X_{i}\to Y_{i}.$$

where $X_{i}$ is the input space and $Y_{i}$ the output space of the component. The rendering node behaves as a passive observer over the outputs from 𝒮, defined as

(3) $$v_{\mathrm{RTV}}:\prod_{k\in 𝒮}Y_{k}⟶R^{3}\times C\times 𝒟,$$

with

- $R^{3}$: spatial transformations (e.g., translation, rotation, scaling);
- $C\subseteq {\left[0,1\right]}^{n}$: extended RGBA and material attributes, including Phong lighting parameters such as ambient, diffuse, and specular reflectance coefficients, shininess exponent, and emission intensity;
- 𝒟: topological mesh modifications (e.g., pointer redirection, vertex displacement, symbolic deformation rules for generalized faults).

For each component $v_{k}\in 𝒮$, a visual mapping function $M_{k}:Y_{k}\to R^{3}\times C\times 𝒟$ is defined, transforming its output into renderable attributes. At each simulation cycle, the render node computes:

(4) $$v_{\mathrm{RTV}}\leftarrow M_{k}\left(h_{k}\left(x_{k}\left(t\right)\right)\right)=\left(\delta_{k}\left(t\right),\mu_{k}\left(t\right),d_{k}\left(t\right)\right),$$

where $x_{k}\left(t\right)$ is the internal state, $h_{k}$ is the output function, and the triple $\left(\delta_{k},\mu_{k},d_{k}\right)$ denotes geometric, visual, and topological transformations, respectively.

This formulation abstracts the rendering logic while preserving modularity. The mapping functions $M_{k}$ are customizable and can reflect continuous, discrete, or symbolic state evolutions. The inclusion of physically grounded illumination parameters, such as those defined by the Phong reflection model, enhances visual realism and supports perceptual evaluation. This passive yet coherent integration supports visual debugging, perceptual model training, and automated dataset generation without altering the physical simulation pipeline.

Moreover, the graph induces a deterministic, topologically ordered schedule and supports incremental recomputation: when a state or parameter changes, only the affected subgraph is evaluated. This yields scalable execution and clear reproducibility, since nodes and connections are fully specified via configuration (parameters and wiring are explicit and versionable). The structure aligns naturally with RITL: perception nodes consume physically consistent states without modifying the physical pipeline.

### 3.3. Lighting and Environmental Control

A realistic representation of lighting and atmospheric conditions is essential for simulations involving visual perception or sensor performance evaluation in outdoor environments. This work implemented a dynamic control mechanism for sunlight and atmospheric sky based on real astronomical models and integrated it into the Gazebo simulator through a custom plugin.

#### 3.3.1. Architecture and Integration

The system architecture at this stage consists of two main modules: (i) an external script, responsible for calculating the solar position and environmental conditions based on geolocation and accelerated virtual time; and (ii) a plugin for Gazebo, which receives the data via TCP socket and updates the directional light (sun) and the simulated sky (sky).

#### 3.3.2. Solar and Atmospheric Model

The solar position is calculated in real time from the site latitude and longitude using the Astral 3.2 Python library [37]. The sunrise and sunset instants are obtained for a given local time instant *t*, denoted by $t_{\mathrm{sunrise}}$ and $t_{\mathrm{sunset}}$. The normalized daytime fraction is then defined as

(5) $$\tau \left(t\right)=\frac{t-t_{\mathrm{sunrise}}}{t_{\mathrm{sunset}}-t_{\mathrm{sunrise}}}.$$

Solar elevation is approximated by

(6) $$\theta_{\mathrm{sun}}\left(t\right)=\sin\left(\pi \cdot \tau \left(t\right)\right)\cdot 90^{\circ }.$$

With this angle, the direct solar irradiance incident on a horizontal surface is estimated using

(7) $$G\left(t\right)=\left\{\begin{array}{cc} G_{0}\cdot \exp\left(-\frac{\tau_{\mathrm{atm}}}{\sin(\theta_{\mathrm{sun}})}\right)\cdot \sin\left(\theta_{\mathrm{sun}}\right), & \mathrm{se}\;\theta_{\mathrm{sun}}>0\\ 0, & \mathrm{otherwise}, \end{array}\right.$$

where $G_{0}=1361\;{W/m}^{2}$ is the solar constant and $\tau_{\mathrm{atm}}$ is the atmospheric turbidity coefficient.

These values are sent to the plugin, which updates the position of sunlight in the XZ-plane, adjusts its direction (up-down during the day and inverted at night), and sets its intensity based on G(t). In addition, the plugin dynamically modifies sky parameters, including relative humidity, wind speed and direction, and visual properties of clouds.

#### 3.3.3. Real-Time Update

Communication between the external script and the plugin occurs in real-time with an update rate of 1 Hz via a TCP connection. Each transmitted packet includes solar angles, light intensity, and atmospheric parameters immediately applied to the Gazebo virtual environment. This mechanism allows the simulation of day–night transitions, slight weather variations, and visual effects consistent with the geographic position of the modeled substation.

Adopting this model not only increases the fidelity of the simulation but also enables experiments sensitive to lighting and weather, which are essential for developing visual perception strategies, automated inspection, and testing of optical sensors under varied conditions.

#### 3.3.4. Perception-Oriented Camera Modeling

To more accurately simulate the optical effects perceived by sensors embedded in mobile robots, a custom camera model was developed, inspired by the properties of the Samsung S23 Ultra lens. The system enables dynamic control of zoom, adjusts the field of view (FOV) according to the operation profile, and reproduces optical phenomena such as distortion and depth-of-field variation.

A dedicated plugin, based on the SensorPlugin class, interacts directly with CameraSensor devices and allows runtime remote zoom control via a ZeroMQ interface. The zoom factor *z* can range from 1× to 100×, with values z≤10 interpreted as optical zoom and z>10 as digital zoom.

In both cases, the horizontal field of view is updated according to

(8) $$\mathrm{FOV}\left(z\right)=\frac{{\mathrm{FOV}}_{\mathrm{base}}}{z},$$

where ${\mathrm{FOV}}_{\mathrm{base}}=1.047$ rad is the default angular aperture of the lens.

For digital zoom levels (z>10), additional adjustments simulate the loss of depth of field by modifying the clipping planes:

(9) $$\begin{array}{c} d_{\mathrm{near}}\left(z\right)=0.1+0.01\left(z-10\right), \end{array}$$

(10) $$\begin{array}{c} d_{\mathrm{far}}\left(z\right)=1000-10\left(z-10\right). \end{array}$$

These parameters are applied dynamically, enabling the simulation of perceptual distortions in environments with significant depth variation.

To complement this perceptual modeling, we incorporated the LensFlareVisualPlugin and parameterized it based on the lighting conditions in the scene. The joint use of active zoom control, dynamic FOV adjustment, and optical effects increases realism for the validation of computer-vision algorithms under challenging conditions (e.g., direct solar glare, high contrast).

## 4. Results and Discussion

**Figure 7:**
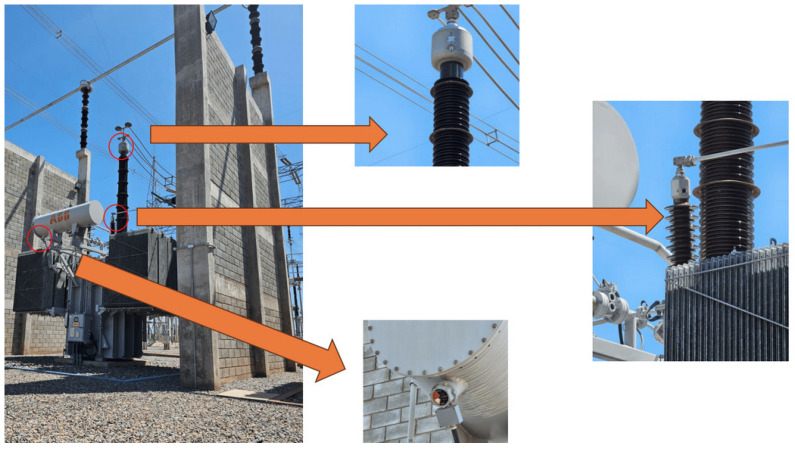
Analog oil level indicators at a shunt reactor.

This section presents a case study designed to validate the proposed simulation framework and demonstrate its applicability to the visual inspection of electrical substation components. The focus is on a shunt reactor equipped with three analog oil level indicators, one frontal and two located on the bushing extensions, as illustrated in Figure 7. These indicators are commonly used in field inspections to monitor the internal condition of the reactor insulation system.

The simulation scenario replicates a typical substation, geometry, materials, and layout, with photorealistic rendering. Sensor modeling uses an RGB-camera model equivalent to a Galaxy S23 but remains device-agnostic via configurable parameters. The case study comprises three stages: (i) formal integration of reactor oil-level indicators into the graph-based framework; (ii) evaluation under ideal visibility/lighting; and (iii) assessment of behavior and perceptual consistency under adverse lighting (e.g., low sun angles, lens flare).

### 4.1. Mathematical Modeling of Oil Level Indicators

**Figure 8:**
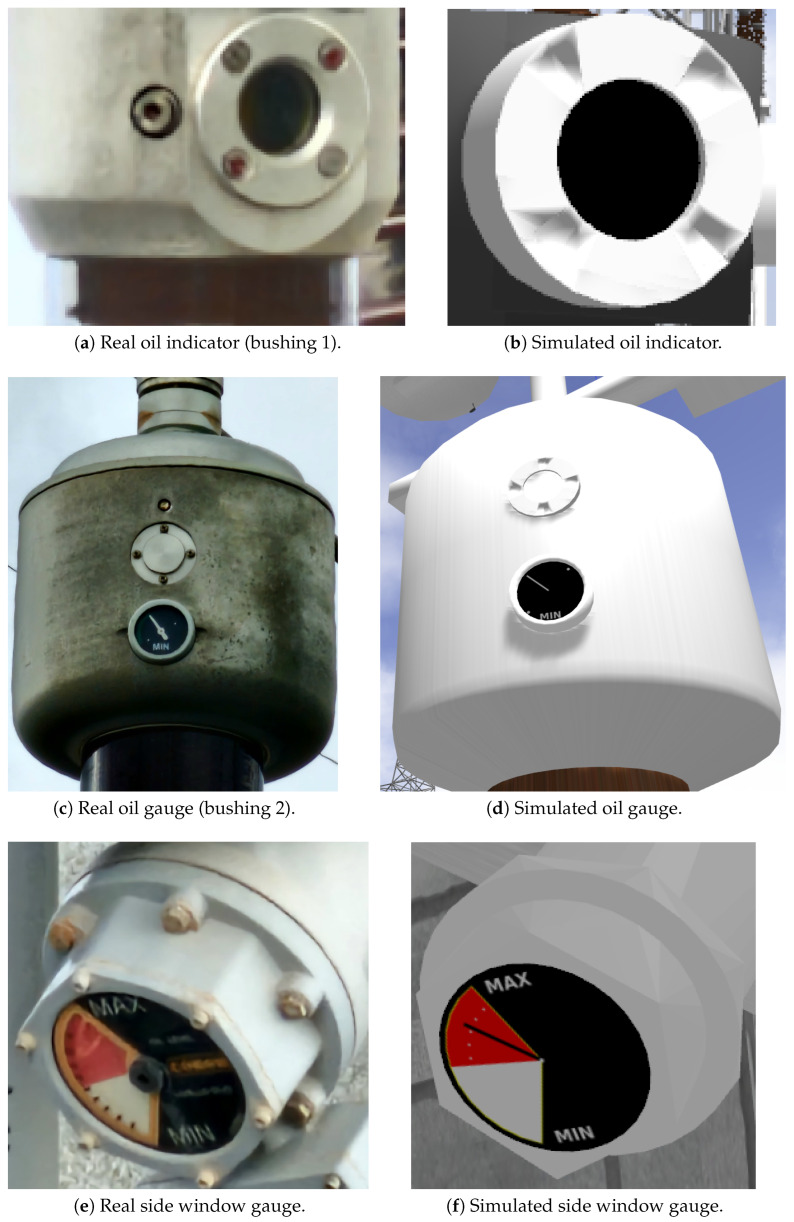
Comparison of oil level indicators in the reactor: real-world images (**left**) and simulated models (**right**).

Figure 8 shows the three oil level indicators of a line reactor. These passive visual gauges are critical for maintenance and inspection routines, offering complementary information about the reactor’s internal condition. The left column displays the real-world images, while the right column presents simulated models developed in the proposed framework.

For this case study, within the proposed graph-based framework, each physical subsystem is represented by a node $v_{k}\in 𝒮\subseteq V$, whose internal dynamics can be described by a state vector of the form:

(11) $$x_{k}\left(t\right)=\left[\begin{array}{c} T_{\mathrm{oil}}\left(t\right)\\ V_{\mathrm{oil}}\left(t\right)\\ L_{b1}\left(t\right)\\ L_{b2}\left(t\right)\\ L_{\mathrm{insp}}\left(t\right) \end{array}\right],$$

where

- $T_{\mathrm{oil}}\left(t\right)$: oil temperature;
- $V_{\mathrm{oil}}\left(t\right)$: oil volume;
- $L_{b1},L_{b2}$: levels observed in the bushings;
- $L_{\mathrm{insp}}$: level in the side inspection window.

In this work, we assume that the thermal insulation properties of the tank and bushings limit the direct influence of daily temperature fluctuations on oil level readings. Under this assumption, significant variations in $L_{b1},L_{b2},L_{\mathrm{insp}}$ are more likely to indicate

- loss of oil mass, caused by leakage, evaporation, or aging effects;
- perturbations in electric field distribution or internal pressure that influence oil displacement.

The oil volume is modeled as

(12) $$V_{\mathrm{oil}}\left(t\right)=V_{0}\left[1+\beta_{T}\left(T_{\mathrm{oil}}\left(t\right)-T_{0}\right)\right]-\Delta V_{\mathrm{loss}}\left(t\right).$$

Here, $V_{0}$ represents the nominal oil volume at baseline temperature $T_{0}$, $\beta_{T}$ is the thermal expansion coefficient of the insulating fluid, and $\Delta V_{\mathrm{loss}}\left(t\right)$ models cumulative oil losses.

In this case study, the output of interest is defined as

(13) $$y_{k}\left(t\right)=h_{k}\left(x_{k}\left(t\right)\right)=\left[\begin{array}{c} L_{b1}\left(t\right)\\ L_{b2}\left(t\right)\\ L_{\mathrm{insp}}\left(t\right) \end{array}\right]\in Y_{k}.$$

The readings are obtained from distinct regions of the reactor and are visualized through either direct-view indicators, based on oil column visibility, or mechanical gauges, which use pointer deflection to indicate level.

Each of these devices is mapped to a visual output by

(14) $$M_{k}:Y_{k}\to C\times 𝒟.$$

with

- $C\in R^{n}$: visual attributes defined by the Phong reflection model, including parameters such as diffuse, ambient, specular, and emissive components.
- 𝒟: mesh-level transformations, including level excursion or pointer deflection for gauges.

At each simulation step, the rendering node passively updates according to

(15) $$v_{\mathrm{RTV}}\leftarrow M_{k}\left(y_{k}\left(t\right)\right)\;\forall v_{k}\in 𝒮.$$

The resulting visual attributes and mesh transformations computed via $M_{k}$ are materialized through Python scripts operating within the Blender API. These scripts translate the abstract rendering directives encoded in $v_{\mathrm{RTV}}$ into concrete updates to geometry, material properties, and texture layers. Once exported, the modified assets are integrated into the Gazebo simulation environment using standard model description formats, ensuring that the RITL mechanism reflects the current internal state of the simulated system with perceptual fidelity.

**Remark** **1.** *Although the state representation and the volumetric model in Equation (12) provide a physically grounded basis for describing oil behavior, the present case study adopts a simplified percentage-based approximation for level variation, as described in the next subsection. This choice allows for fast and interpretable simulation of visual indicators while remaining conceptually compatible with the thermodynamic assumptions underlying the framework.*

The following sections present simulation results under both ideal and non-ideal lighting conditions, aimed at evaluating the visual coherence and robustness of the rendering pipeline. All experiments were conducted on a machine running Ubuntu 20.04 with Gazebo 11 and Blender 4.4, using an Intel Core i7 processor (3.40 GHz), 32 GB of RAM, and an NVIDIA GeForce RTX 2060 GPU with 6 GB of VRAM.

### 4.2. Simulation Under Ideal Lighting Conditions

To evaluate the behavior of the oil level indicators under controlled visual conditions, we conducted a simulation using physically inspired variations of electrical and thermal quantities. The goal was to assess the consistency of the graphical rendering and the realism of dynamic level changes over time.

We intentionally adopted lightweight, phenomenological dynamics to emphasize that the framework allows users to choose the desired model fidelity according to application needs and available computational resources. The same module can be replaced by any physics-based formulation whenever calibrated parameters or measurements are available, without changes to the surrounding pipeline. In particular, a thermofluid backend could be coupled—offline (precomputed traces) or online—to drive oil-level dynamics and temperature-dependent appearance.

The simulation ran for 60 s, with level readings updated every 5 s (0.2 Hz). At each simulation step, three indicators were updated based on the following physical considerations:

- Main tank level: The main tank oil level decreases linearly at a rate of 1% per second until reaching a minimum threshold of 25%, representing oil loss due to leakage or degradation. A temperature-dependent factor introduces a slight thermal expansion effect based on the ambient temperature $T_{\mathrm{env}}=35\;{}^{\circ}C$:
  $${\mathrm{level}}_{\mathrm{tank}}\left(t\right)=\min\left(100.0,\;\max\left(25.0,\;100.0-1.0\cdot t\right)\cdot (1+0.001\cdot \left(T_{\mathrm{env}}-25\right))\right).$$
  This approximation preserves the qualitative behavior of volume variation while simplifying the simulation.
- Bushing levels: The oil levels in the bushings were generated using a phenomenological model combining oscillatory behavior, synthetic load signals, and bounded noise. Although time-varying current and voltage expressions were used:
  i(t)=200+50·sin(0.3·t),v(t)=13800+500·cos(0.4·t),
  They were not derived from physical equations but rather serve to modulate the base level:
  base(t)=50+0.08·i(t)+0.005·(v(t)−13800)
  A dynamic component and noise were added to simulate internal variability:
  $${\mathrm{level}}_{\mathrm{bushing}}\left(t\right)={\mathrm{clip}}_{\left[30,\;100\right]}\left(\mathrm{base}(t)+25\cdot \sin(0.25\cdot t+\phi )+\xi \right),$$
  where ϕ is a phase offset (in radians), and ξ∼U(−2.0,2.0) represents uniform noise.
- Phase offset: A constant delay of Δt=1.2s was introduced between the bushing indicators to simulate asynchronous behavior across different components:
  $${\mathrm{level}}_{\mathrm{bushing}2}\left(t\right)={\mathrm{level}}_{\mathrm{bushing}1}\left(t+\Delta t\right).$$

Each value, expressed as a percentage from 0% to 100%, was passed as an argument to Blender export scripts responsible for generating updated visual assets. These scripts controlled the visible fluid levels in the simulated gauges, ensuring visual fidelity aligned with the physical model.

**Figure 9:**
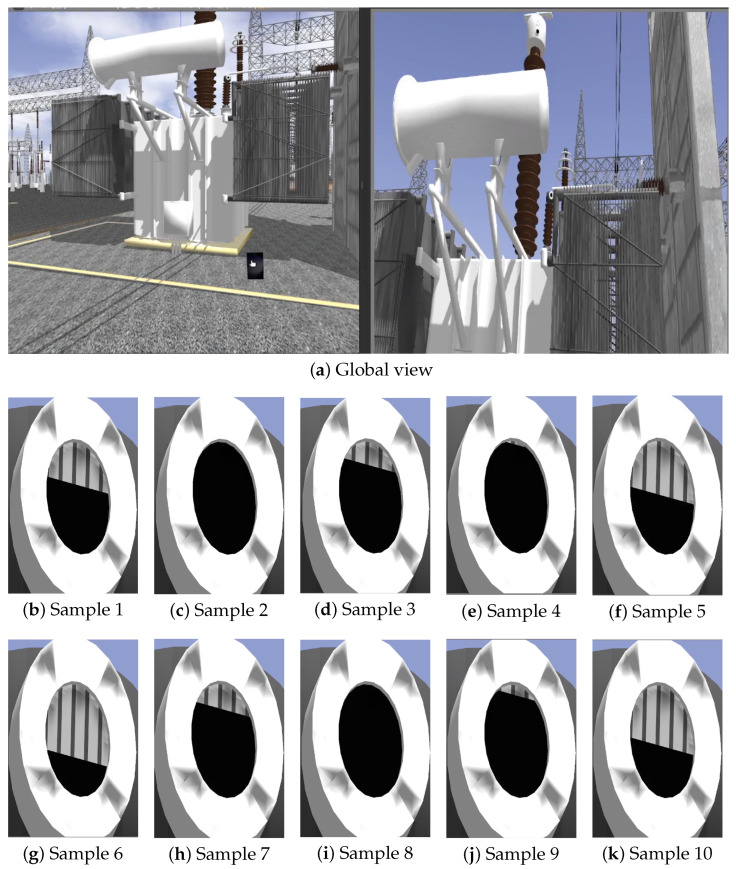
(**a**) Global view of the indicator component—left: sensor positioning; right: unzoomed gauge view. (**b**–**k**) Close-up crops at 45× zoom showing simulated gauge readings with varying oil levels.

**Figure 10:**
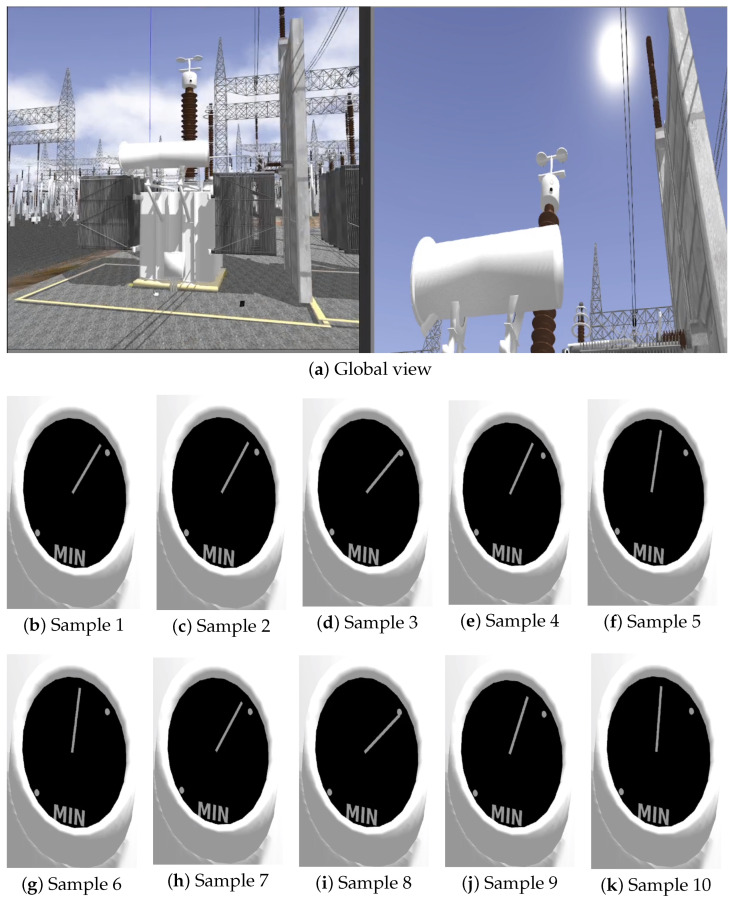
(**a**) Global view of the indicator component—left: sensor positioning; right: unzoomed view of the gauge. (**b**–**k**) Individual test samples at 45× zoom showing simulated gauge readings with varying oil levels.

**Figure 11:**
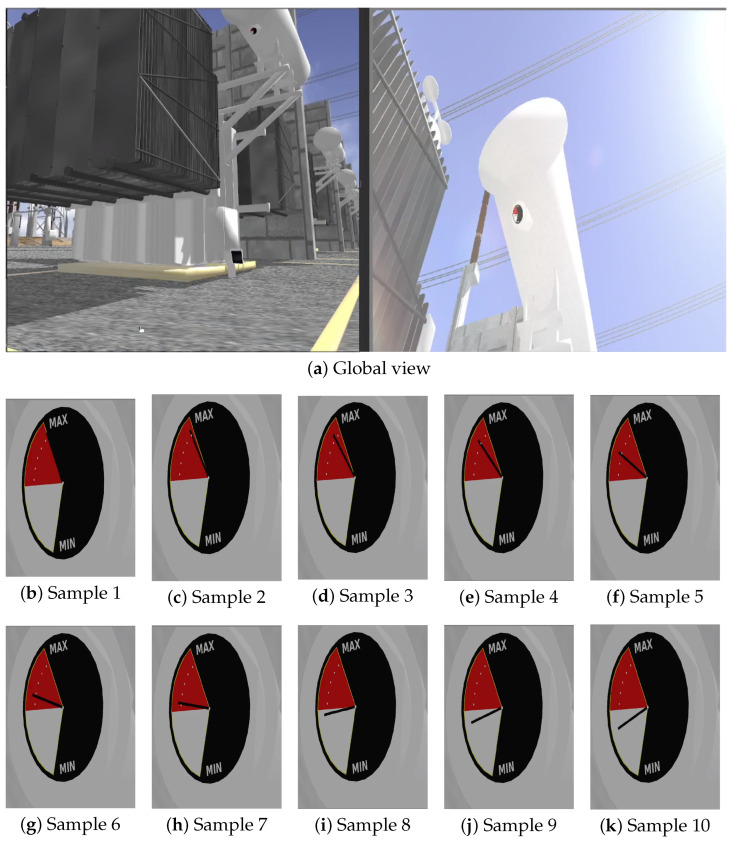
(**a**) Global view of the indicator component—left: sensor positioning; right: unzoomed view of the gauge. (**b**–**k**) Detailed views at 45× zoom illustrating simulated gauge readings with varying oil levels.

Figure 9, Figure 10 and Figure 11 illustrate selected frames from the simulation, showing dynamic variations in fluid levels across all three indicators under ideal lighting.

In the global scenario images, the left panel shows the visual sensor (Samsung Galaxy S23 Ultra) oriented toward the object of interest, and the right panel presents the unzoomed camera image. The smaller images illustrate samples of the region of interest after a 45× zoom is applied. Although rendered under ideal lighting, slight visual variations can already be observed depending on the camera pose and orientation, a factor further explored in the next section.

This setup demonstrates the capability of the proposed framework to represent real-time variations caused by gradual degradation, thermal expansion, and electrical perturbations, without relying on external lighting inconsistencies, thus isolating visual effects from perceptual dynamics. Moreover, the rendering remains consistent across different conditions, enabling controlled evaluation of computer vision algorithms under repeatable and physically meaningful scenarios.

However, while ideal lighting ensures that visual changes stem solely from physical dynamics, variations in camera pose relative to the sun begin to introduce optical phenomena such as lens flare and light scattering, as seen in later simulation frames. Although visually realistic, these artifacts can significantly affect perceptual interpretation and algorithmic processing. The following section thoroughly explores this influence, analyzing the interaction between lighting conditions and the robustness of rendered visual cues.

### 4.3. Simulation Under Adverse Lighting Conditions

This case study evaluates the ability of the RITL simulation framework to produce visually coherent outputs of oil level indicators under varying natural lighting conditions. Each indicator is rendered using photometric material properties and dynamically updated to reflect different oil volumes, according to the visual mapping function previously formalized.

**Figure 12:**
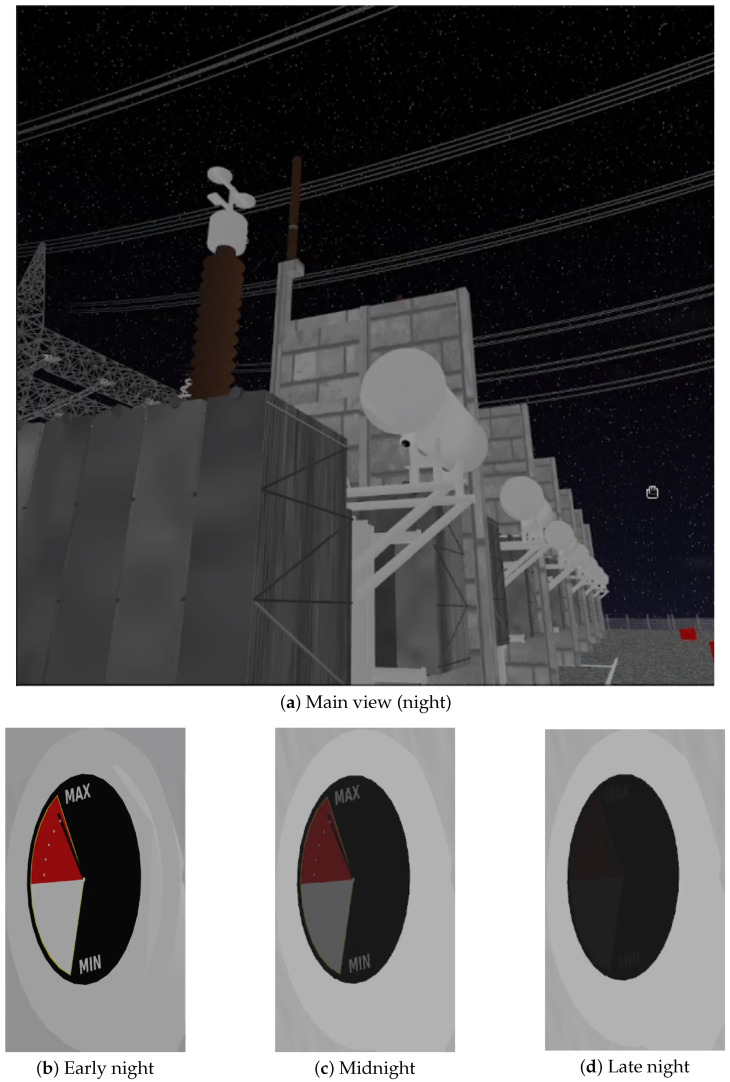
Scenario 1—Night lighting: (**a**) Overview of the indicator. (**b**–**d**) Close-up views highlighting key features.

**Figure 13:**
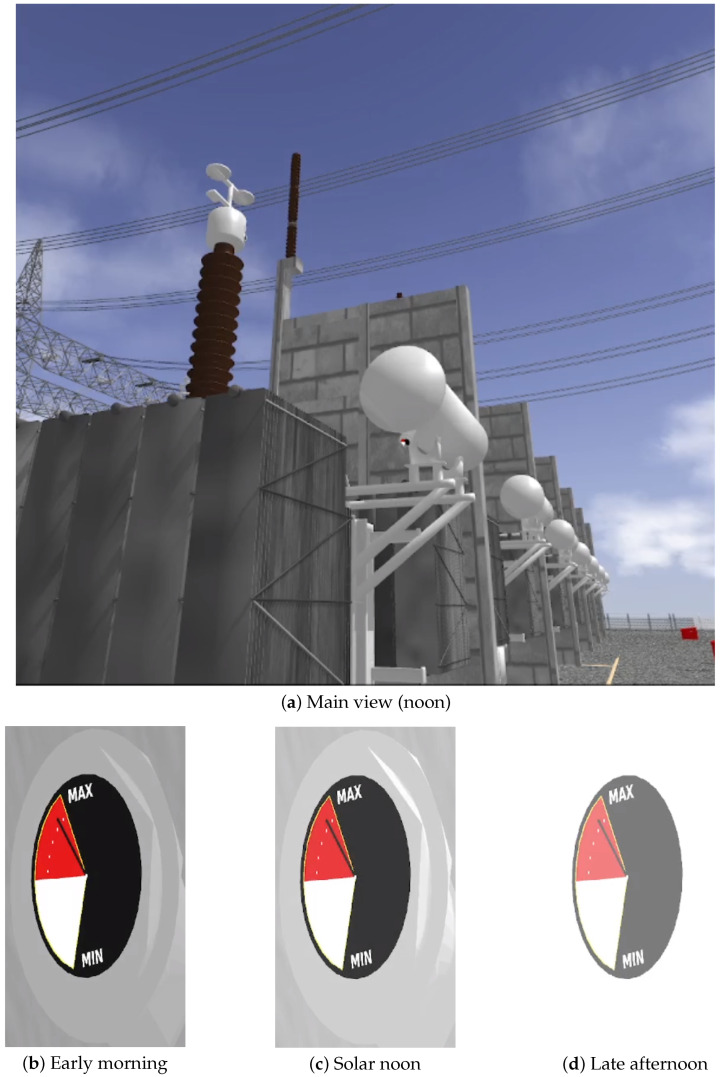
Scenario 2—Noon lighting: (**a**) Overview of the indicator. (**b**–**d**) Close-up views highlighting key features.

To assess the robustness of visual perception under varying illumination conditions, the main tank indicator was simulated at three distinct time windows during both night and day periods. Figure 12 and Figure 13 illustrate the complete view of the scene alongside close-up visualizations of the indicator for each lighting condition.

In the night scenario (Figure 12), the indicator exhibits significant visual degradation due to low illumination. The three detail views (b–d) show progressive changes in visibility and contrast, simulating temporal transitions such as early night, midnight, and late night. In some frames, only the circular silhouette of the indicator is distinguishable, with minimal color distinction.

In contrast, the day scenario (Figure 13) presents the same viewpoints under increasing daytime illumination levels, roughly corresponding to early morning, noon, and late afternoon.

The main scene receives increasing sunlight across the simulated daytime intervals. While early frames enhance visibility through stronger illumination and color definition, the last close-up reveals signs of oversaturation, with washed-out highlights that slightly compromise visual clarity.

These results demonstrate the framework’s capability to reproduce perception-relevant visual phenomena under a range of realistic lighting dynamics, supporting the development and validation of computer vision strategies for substation inspection.

To quantify the progressive visual degradation of the gauge images caused by varying illumination throughout the simulated time windows, three perceptual metrics were computed directly from the zoomed-in indicator views:

- RMS Contrast C∈[0,1], measuring global contrast based on the standard deviation of grayscale pixel intensities, normalized by the maximum possible value:
  (16) $$C=\frac{\sqrt{\frac{1}{N}\sum_{i=1}^{N}{\left(I_{i}-\bar{I}\right)}^{2}}}{255}$$
  where $I_{i}$ is the intensity of pixel *i*, $\bar{I}$ is the image mean intensity, and *N* is the total number of pixels.
- High-Intensity Ratio S∈[0,1], representing the proportion of near-saturated pixels with intensity above 240, used to detect overexposed regions:
  (17) $$S=\frac{1}{N}\sum_{i=1}^{N}I\left[I_{i}>240\right]$$
- Visible Oil Height Ratio *H*, which quantifies the vertical extent of the dark pointer inside the colored gauge region. A fixed ROI (region of interest) around the gauge is converted to HSV. The gauge region is segmented as the union of (i) a red mask (hue H∈[0°,10°]∪[160°,180°], saturation S≥100, value V≥50) and (ii) a white mask (low saturation S≤60, high value V≥200). The pointer is then detected as dark pixels inside this region using a black mask (H∈[0°,180°],S∈[0,255],V≤80). Let $y_{\min}$ and $y_{\max}$ be the top/bottom row indices of the detected pointer pixels; we define
  $$H\;=\;\frac{y_{\max}-y_{\min}}{H_{\mathrm{ref}}},$$
  where $H_{\mathrm{ref}}$ is the pointer height measured in the first image of each test sequence under similar illumination. All thresholds are fixed across all sequences to avoid tuning bias.

**Remark** **2.** *Robustness. Using HSV masks makes the segmentation less sensitive to moderate color changes, and the dark-pointer-vs-bright-fill design reduces dependence on chroma shifts. Specular highlights are not explicitly removed in this study; to contextualize frames with possible glare, we also report the proportion of near-saturated pixels (S: fraction of grayscale intensities > 240). Strong, localized glare overlapping the pointer may underestimate H, which we note as a limitation.*

These metrics enable an objective assessment of how visual fidelity is affected across lighting conditions and time intervals, particularly in terms of contrast loss, overexposure, and loss of perceptual cues such as fluid level visibility.

Table 1 summarizes the results across six gauge images from both scenarios shown in Figure 12 and Figure 13.

These results demonstrate that, despite significant lighting variations, the simulated oil level indicators exhibit perceptual consistency in most views. While contrast (*C*) values remain above 25% in the majority of gauge crops, saturation artifacts (*S*) increase substantially under noon lighting conditions—reaching values above 70% in some close-ups (e.g., Figure 13d). This behavior is expected in overexposed regions, yet the oil height ratio (*H*) is preserved in key views (e.g., Figure 12b and Figure 13c), ensuring functional visibility.

These findings validate the effectiveness of the proposed RITL rendering mechanism for perception validation tasks, even under adverse illumination scenarios; a video walkthrough of the results is available [38].

**Remark** **3.** *Limitation. Our analysis uses fixed ROIs and global thresholds across sequences; strong localized glare may underestimate H, and noon overexposure compresses dynamic range, affecting C and inflating S.*

At the framework level, this paper concentrates on the sensor-centric vision layer and image-level characterization; task-level benchmarks, quantitative radiometric/sky-model calibration, and additional modalities (e.g., thermal, LiDAR) are outside the scope. System performance is hardware/scene dependent, so platform-specific FPS tables are omitted.

The case study covers one representative asset, and full assets are not publicly released due to sponsor confidentiality, although the procedure is reproducible with independent models. These are scope choices rather than hard constraints—the graph-based, modular design supports drop-in calibration, new sensors, and task-level studies, which we plan to report in follow-on work.

## 5. Conclusions and Future Work

This paper presents a sensor-centric digital twin simulation framework for vision in electrical power substations, combining accurate 3D geometry with physically based lighting dynamics (realistic diurnal variation, interactive sun-pose control), and representative optical imperfections in an RITL pipeline.

The proposed architecture supports configurable sensor models, dynamic object properties, and a programmatic diurnal cycle with controllable sun pose (azimuth/elevation) and irradiance, enabling the reproduction of key perceptual challenges observed in real-world inspections.

Validation experiments demonstrate the ability of the framework to capture time-dependent visual effects, including object changes at runtime and material responses under variable lighting. These capabilities support developing, testing, and benchmarking robotic perception algorithms in complex, large-scale environments.

A key contribution of this work is the integration of real-time rendering tied to the internal state of the simulation, enabling visual feedback that includes geometric transformations, appearance changes, and mesh-level modifications to simulate diverse conditions. The graph modeling formalism represents each physical subsystem as a node with internal dynamics. In contrast, linked perceptual nodes coordinate the generation of visuals consistent with the internal state. Although this study focused on representative components such as oil-level gauges, the same principles extend to other typical substation elements, including insulators, disconnect switches, and metal structures, reinforcing the potential of the approach for autonomous inspection and perception validation.

The framework is useful to utilities and asset owners, robotics/computer-vision researchers, and inspection/sensor vendors for reproducible dataset generation, benchmarking, and pre-deployment testing.

Future work will focus on expanding the expressiveness of deformation models, refining material dynamics under temporal stimuli, and integrating additional sensor modalities. Another ongoing direction is the automation of dataset generation workflows to support deep learning pipelines and evaluate perceptual models under diverse and reproducible conditions.

## Figures and Tables

**Table 1:** Quantitative visual analysis for gauge regions under varying lighting conditions.

Gauge View (Figure)	Contrast *C*	Bright Pixels *S*	Normalized Height *H*
Early night (Figure 12b)	0.2634	0.00000	1.00
Midnight (Figure 12c)	0.2510	0.00000	0.66
Late night (Figure 12d)	0.2698	0.00000	0.00
Early morning (Figure 13b)	0.2579	0.05562	1.00
Solar noon (Figure 13c)	0.2502	0.06729	0.90
Late afternoon (Figure 13d)	0.2087	0.74833	0.00

## Data Availability

The data that have been used are confidential.

## Abbreviations

The following abbreviations are used in this manuscript:

DT	Digital Twin
RITL	Render-In-The-Loop
ROI	Region of Interest
HSV	Hue–Saturation–Value (color space)
RMS	Root Mean Square
FOV/HFOV	(Horizontal) Field of View
PSF	Point Spread Function
SDF	Simulation Description Format
PTZ	Pan–Tilt–Zoom (camera)
CAD	Computer Aided Design
DAE	Digital Asset Exchange
LOD	Level of Detail
FPS	Frames Per Second
RTF	Real-Time Factor
PBR	Physically Based Rendering
CFD	Computational Fluid Dynamics
API	Application Programming Interface
